# Feasibility and acceptability of novel methods to estimate antiretroviral adherence: A longitudinal study

**DOI:** 10.1371/journal.pone.0210791

**Published:** 2019-01-15

**Authors:** Parya Saberi, Kristin Ming, Dominique Legnitto, Torsten B. Neilands, Monica Gandhi, Mallory O. Johnson

**Affiliations:** 1 Department of Medicine, Division of Prevention Sciences, University of California San Francisco, San Francisco, CA, United States of America; 2 Department of Medicine, Division of HIV, Infectious Diseases, and Global Medicine, University of California San Francisco, San Francisco, CA, United States of America; Brown University, UNITED STATES

## Abstract

Due to marked reductions in morbidity and mortality, antiretroviral (ARV) adherence monitoring is of high interest. Researchers and clinicians often resort to the most feasible and cost-effective adherence methods possible, which may result in biased or inaccurate estimates and require the physical presence of a participant at a research or clinic site. The objective of our study was to evaluate the feasibility and acceptability of three objective, innovative, and remote methods to estimate ARV adherence which may be conducted with less time and financial resources in a wide range of clinic and research settings. These three methods included: (a) text-messaged photographs of pharmacy refill dates to measure refill-based adherence, (b) text-messaged photographs of ARV medications to estimate pill-count-based adherence, and (c) home-collected hair samples for the measurement of ARV concentration to determine pharmacologic-based adherence. We conducted a pilot study from March through October 2017 to examine the feasibility and acceptability of these three adherence measures and the remotely conducted study procedures in 93 adults living with HIV nationwide. From our diverse national sample of participants, 95.7% were retained until the end of the study, 89.9% sent all text messages, and 84.3% sent all hair samples. Approximately 74.2% of participants reported excellent overall experience with the study, 60.2% were very or extremely satisfied with participating in a hair collection study, and 76.3% noted extremely high likelihood of participating in a similar study including text messaging pictures of medications or refill dates. We noted high levels of feasibility and acceptability with the remote study methodology, collection of photographed and text messaged pharmacy refill dates and pill counts, and home-collected hair samples. Here we describe the feasibility and acceptability metrics, results from the exit qualitative interviews with the participants, and lessons learned. These adherence measures represent innovative approaches to expand monitoring tools for HIV treatment and prevention adherence in future research.

## Introduction

Adherence to antiretroviral (ARV) medications is the primary determinant of virologic suppression, leading to marked benefits on morbidity and mortality from HIV[1, 2], but long-term adherence is difficult to maintain. Adherence monitoring is thereby of increasing interest; however, there is no gold standard for estimating adherence, as all methods offer opportunity for bias or inaccuracy[3]. Current methods include the use of patient self-report[4, 5], pill counts[4], pharmacy refill dates[6, 7], electronic drug monitoring (such as MEMS caps[4, 8] or Wisepill[9]), and pharmacologic measures (such as concentrations in blood[10] or hair[11]). Each method has varying advantages and disadvantages and the choice of which to use often depends on feasibility and cost-effectiveness. For example, self-report is inexpensive, but subject to a host of reporting biases that can result in an overestimation of adherence. Electronic medication monitoring systems, such as MEMS or Wisepill, can provide granular, daily data reflecting detailed adherence patterns. However, the devices measure medication bottle/device opening only (not actual ingestion) and are expensive. Furthermore, some individuals may prefer not to carry them for their HIV medications, as the bottles/devices are often large and conspicuous, thus potentially inviting attention and activating HIV-related stigma regarding the purpose of the medication. Feasible, acceptable, cost-effective, and objective measures of ARV medication adherence are needed to minimize biased or inaccurate estimates.

The vast majority of HIV adherence research to date has required the physical presence of a participant at a research or clinical care site. Transportation costs, time constraints, and potential stigma associated with participation in research related to HIV[12] may all lead to people living with HIV (PLWH) declining or discontinuing participation in research protocols. The consequence of such barriers can be the limited generalizability of research findings, missing data, or potentially biased results. Remotely conducted research, where an individual can participate without location or time restriction, may be a potential solution to minimizing barriers related to transportation, time, and stigma.

In this study, we examined the feasibility and acceptability of three innovative methods to estimate ARV medication adherence that were all implemented using remotely conducted study procedures. These methods included text messaged photographs of pharmacy refill dates, text messaged photographs of pills, and home-collection of hair samples. These three metrics were selected due to their capacity to be collected remotely, time efficiency for study staff and participants, objectivity, and relative cost-effectiveness.

## Methods

### Study overview and design

From March through October 2017, we conducted a pilot study to examine the feasibility and acceptability of three novel methods of ARV adherence assessment using (a) text-messaged photographs of pharmacy refill dates to measure refill-based adherence, (b) text-messaged photographs of ARV medications to estimate pill-count-based adherence, and (c) home-collected hair samples for the measurement of ARV concentration to determine pharmacologic-based adherence. We further determined the feasibility and acceptability of a study where all study activities—including recruitment, consent, compensation, hair sample collection, text messaging, and exit interviews—were conducted remotely. The study protocol has previously been published and includes detailed study procedures[13]. We received approval from the University of California, San Francisco (UCSF) Institutional Review Board to conduct this study and all participants provided electronic informed consent.

Inclusion criteria for the study included age ≥ 18; HIV infection; taking a stable ARV regimen for at least three consecutive months prior to participation containing tenofovir (either tenofovir disoproxil fumarate [TDF] or tenofovir alafenamide [TAF]), emtricitabine (FTC), darunavir (DRV), or dolutegravir (DTG); access to a mobile telephone that allowed photography and text messaging of photographs; and access to the internet. Exclusion criteria included receiving automated ARV refills; receiving renally-dosed ARV medications due to chronic kidney disease; and inability to provide a hair sample (e.g., due to baldness). Recruitment was conducted online using social media (e.g., Facebook and Twitter) and via flyers at local clinics, emails, and snowball sampling.

### Study procedures

Links for Qualtrics surveys (version 2017, Provo, UT, USA), a data collection software program used to conduct online surveys, were emailed to participants to obtain informed consent, collect initial demographic information (age, sex, gender, race, ethnicity, sexual identity, financial situation, education, incarceration, homelessness, and first three digits of zip code), clinical data (HIV Viral Load and CD4+ cell count), ARV information (ARV name, dosing frequency), and conduct the exit survey.

At baseline and monthly, for six months, study staff communicated via bi-directional text messages with participants to text message their self-reported adherence using the adherence rating item[14], text-message photographs of their pharmacy refill date, and text-message photographs of their remaining pills for pill count. At baseline and every two months, for six months, participants were mailed a hair collection kit and asked to mail back a hair sample. Participants were compensated up to a maximum total of US$270 for completing all study activities.

Hair concentrations of ARVs were measured in the UCSF Hair Analytical Laboratory (HAL) using validated liquid chromatography-tandem mass spectrometry (LC-MS/MS) based methods. These methods have been peer-reviewed and approved by the National Institute of Health’s Division of AIDS’ Clinical Pharmacology and Quality Assurance Program[15].

### Study outcomes

The feasibility of conducting this study was evaluated by the study’s ability to enroll, retain, and engage participants until the completion of the study and by pre-specified metrics: (a) mean length of time to recruit participants; (b) percentage engaged until the end of study; (c) mean frequency of non-response (i.e., participant failure to send text messages); (d) mean number of text messages where clear photographs were received with the required information; (e) mean number of days between the date of request for photographs and the participant’s response; (f) mean number of reminder text messages prior to participant’s response; and (g) mean number of hair samples that participants mailed back to the study. The pre-specified metrics were calculated based on total enrolled at baseline. The definition and acceptance criteria for these metrics are listed in the Pre-specified Feasibility Metrics table and further discussed in the study protocol paper[13]. Finally, we examined a range of feasibility metrics that were not selected *a priori* and were added during the course of the study (i.e., post-specified feasibility metrics). These included the number of participants (a) with a stock supply of medications at baseline (which made the calculation of adherence based on refill dates and pill count challenging), (b) showing an increase in pill count without having received a new refill, (c) sending text messages with refill dates not in chronological order, (d) receiving a 60- or 90-day medication supply (which resulted in the receipt of identical refill dates for 2–3 months in a row), (e) unable to find their refill date, (f) sending low volume of hair (the LC-MS/MS process includes the separation of 1–2 mm of the proximal section of the hair sample and weight of 5 mg), and (g) not labeling the distal end of their hair samples (the proximal end of the hair sample is used in the LC-MS/MS process; therefore, the distal end is labeled to distinguish the two ends).

The second study outcome, acceptability, was examined via exit surveys and structured qualitative interviews with participants at six months. The exit survey, a 21-item questionnaire, was conducted with all study participants and included Likert scale questions pertaining to (a) the overall rating of the study; (b) satisfaction with each study procedure (i.e., remotely conducted study, collecting hair samples, mailing hair samples, text messaging photographs of medications and refill dates, security and privacy of the text messages, level of information and staff support, remote receipt of study compensation using a reloadable debit card); (c) ease or difficulty with each study procedures (i.e., collecting hair, using hair kits, mailing hair kits back to the study, taking photographs of medications and refill dates, text messaging photographs of medications and refill dates); (d) helpfulness of communication with study staff, hair collection instruction video, written hair collection instructions; (e) recommending a study similar to this to a friend; and (f) participating again in a similar study. We also asked participants if they received help in collecting their hair, if they collected hair from other places than their head, and if participation in the study changed their medication adherence. Questions were assembled by the study team based on their prior research experience, and sample items include “How would you rate your overall experience with the study?” or “How satisfied were you with participating in a research project that was conducted without you having to come into a clinic or research site (i.e., remotely)?” Responses to questions are detailed in Responses to Exit Survey table.

The exit interviews included structured questions that were asked by study staff over the telephone at six months and lasted approximately 30 minutes (S1 Table). To maximize our understanding of the range of experiences with various study components, interviews were conducted with one-third of participants (a target to attempt to reach saturation of responses within each interview group) who met one of the following pre-specified study engagement categories and were randomly-selected from each category: (a) On Time: individuals who responded to the monthly text messages *and* sent hair samples within a predefined timeframe, i.e., within 5 days after the text messages were requested and 11 days after the hair kit was sent (target N = 12); (b) Early Inconsistent Hair Samples: participants who were consistent with their text messages but sent one or more hair samples up to six days after the predefined timeframe for hair sample, i.e., within 17 days after hair kit was sent (target N = 5); (c) Late Inconsistent Hair Samples: participants who were consistent with their text messages but sent one or more hair samples at least seven days after predefined timeframe for hair samples, i.e., 18 days or later after the hair kit was sent or not at all (target N = 5); (d) Inconsistent Texts: individuals who were consistent with their hair samples but at any point in the study responded to the text messages after the predefined timeframe for text message (i.e., 5 days after the text messages were requested) or did not respond to the text messages at all (target N = 5); and (e) Least Consistent: participants who *both* responded to the text messages and sent their hair samples after the predefined timeframes or not at all (target N = 5).

### Data analysis

Univariate analyses were conducted using Stata (StataCorp. 2015. Version 14. College Station, TX) to compute means, percentages, medians, and standard deviations for quantitative feasibility and acceptability data. Qualitative exit interviews were audio-recorded, transcribed verbatim, and analyzed using a Microsoft Excel matrix where each column corresponds to themes from each qualitative interview question and each row represents a case. This method allowed for the identification of patterns in the distribution of themes for data analysis[16]. One co-author categorized each interview using this matrix. Another co-author double coded a random subsample of seven interviews to ensure consistency of the coding process. Coding discrepancies were discussed by the two authors until consensus was reached or arbitrated by the first author. Once consistency across coders was established, the remaining transcripts were coded by one coder only.

## Results

We screened 166 people nationwide and enrolled 93. Seventy three individuals were ineligible due to not being interested (N = 34); not able or willing to provide hair samples due to baldness, very short hair, or not wanting to cut hair (N = 9); receiving automated ARV refills (N = 9); not taking an ARV regimen from the list of study ARVs (N = 7); having recently changed ARV medications or planning to switch soon (N = 7); HIV-negative serostatus (N = 3); not having access to the internet (N = 2); participating in a blinded clinical trial (N = 1); or being mono-lingual Spanish speaker (N = 1).

Table 1 details the characteristics of the 93 individuals who consented to participate in the study. Four individuals withdrew from the study due to not wanting to provide hair samples (N = 3) and being unable to send photograph text messages despite coaching from study team members (N = 1) and three were lost-to-follow-up. The majority of participants were male (83.9%), White (61.3%), gay identified (68.8%), and had greater than high school education (60.2%). Most participants were geographically located in the Western US (43%). From an HIV clinical care standpoint, most participants reported an undetectable HIV viral load (90.3%), were on once-daily ARVs (93.6%), and just over half (55.9%) reported excellent adherence using the adherence rating item[14].

**Table 1:** Participant characteristics.

Characteristic	Sub-categories	N = 93
Age, mean (SD)		44.4 (1.4)
Sex, N (%)		
	Male	82 (88.2)
	Female	9 (11.8)
	Decline to answer	2 (2.2)
Gender, N (%)		
	Male	78 (83.9)
	Female	9 (9.7)
	Transgender Female	4 (4.3)
	Transgender Male	1 (1.1)
	Genderqueer	1 (1.1)
Race, N (%)		
	White	57 (61.3)
	African American/Black	25 (26.9)
	Multiracial	5 (5.4)
	Asian	2 (2.2)
	Other or decline to answer	4 (4.3)
Latino Ethnicity, N (%)		15 (16.1)
Sexual Identity, N (%)		
	Gay	64 (68.8)
	Heterosexual	16 (17.2)
	Bisexual	7 (7.5)
	Other or decline to answer	6 (6.5)
Financial Situation, N (%)		
	I have enough money to live comfortably	38 (40.9)
	I can barely get by on the money I have	39 (41.9)
	I cannot get by on the money I have	11 (11.8)
	Decline to answer	5 (5.4)
Education, N (%)		
	Less than high school	6 (6.5)
	High school or GED	30 (32.3)
	Greater than high school	56 (60.2)
	Decline to answer	1 (1.1)
Ever Jail, N (%)		20 (21.5)
Ever Homeless, N (%)		32 (34.4)
Geographic Region*, N (%)		
	West	40 (43)
	Midwest	29 (31)
	South	18 (19)
	Northeast	6 (6)
CD4 Cell Count, mean (SD)		664.8 (40.7)
HIV Viral Load, N (%)		
	Undetectable	84 (90.3)
	Detectable	8 (8.6)
	Missing	1 (1.1)
Antiretroviral Frequency, N (%)		
	Once-daily	87 (93.6)
	Twice-daily	6 (6.5)
Adherence Rating, N (%)		
	Excellent	52 (55.9)
	Very good	27 (29.0)
	Good	11 (11.8)
	Fair	3 (3.2)
	Poor	0 (0)
	Very poor	0 (0)

GED: General Equivalency Diploma; SD: standard deviation

* Based on United States’ Census Bureau-designated regions and divisions

### Feasibility

We enrolled 93 individuals in 50 days and retained 95.7% of the participants until their last study encounter (Table 2). At six months, 89.9% of participants responded to all text messages, 84.3% mailed hair samples, and 80.9% sent both all text messages and hair samples. From a total of 538 text message responses that were expected after baseline, we received text messages from 90.1% of participants within five days after the initial request across all time points. Study staff sent a total of 244 text message reminders for participants to respond to study text messages which equated to 2.6 reminders per participant (Table 3). We received no responses to 13 of the 538 text message requests (non-response = 2.4%), which was mainly among the three individuals who were lost to follow-up.

**Table 2:** Feasibility of participant enrollment, engagement, and retention.

Time Point	0	1	2	3	4	5	6
Enrolled/Retained, N (%)	93 (100)	91 (97.8)	90 (96.8)	90 (96.8)	89 (95.7)	89 (95.7)	89 (95.7)
Sent all text messages, N (%)	86 (92.5)	88 (96.7)	86 (95.6)	86 (95.6)	84 (94.4)	82 (92.1)	80 (89.9)
Mailed all hair samples, N (%)	88 (94.6)	.	86 (95.6)	.	78 (87.6)	.	75 (84.3)

**Table 3:** Pre-specified feasibility metrics.

Feasibility Metric	Definition/Acceptance Criteria	Calculation
Length of time to recruit participants	Duration of 3 months	Recruitment duration = 50 days (1.67 months)
Percentage engaged until end of study	Text messaged photographs of refill dates and pill counts at 6 months from at least 80% of enrolled participants	Received text messages at 6 months / enrolled participants = 80/93 (86.0%)
Mean frequency of non-response	A mean of two non-responses per enrolled participant	Non-responses per enrolled participant = 13/93 (0.1)
Mean number of text messages to receive a clear photograph with required information	A mean of two additional text messages per enrolled participant	Clarifying text messages per enrolled participant = 88/93 (0.9)
Mean number of days between the request for text messages and participants’ response	At least 80% of responses within 5 days of first text message request among enrolled participants	Text messages received / text messages expected within 5 days = 485/538 (90.1%)
Mean number of reminder text messages prior to response	A total of two reminder text messages per enrolled participant	Reminders per enrolled participant = 244/93 (2.6)
Mean number of hair samples mailed back to study	Receipt of three out of four mailed hair samples from at least 80% of enrolled participants	Received 3 out of 4 hair samples per enrolled participant = 81/93 (87.1%; mean of 3.5 samples per enrolled participant)

We received a total of 327 hair samples from an expected 361 (90.6%). We completed exit surveys with 94.4% (84/89) of participants and qualitative exit interviews with 97% (32/33) of those randomly selected for our pre-specified engagement categories.

Table 3 summarizes the pre-specified metrics that were used to examine study feasibility based on the total number of participants enrolled (N = 93). All feasibility metrics met the acceptance criteria, except the mean number of reminder text messages prior to response.

For our post-specified feasibility metrics, 39 participants (41.9%) reported having a stock supply of medications at baseline. Nineteen participants had inaccurate pill count data over 22 time points (i.e., showing an increase in pill count without having received a new refill) and five participants sent text messages with refill dates that were not in chronological order. Nineteen individuals received a 60- or 90-day supply and 10 participants reported not being able to find their refill date due to reasons such as discarding the medication box before taking a photograph of the refill date.

There was one hair sample over the entire duration of the study (at month 4) that could not be analyzed due to a low volume of hair. At 0, 2, 4, and 6 months, 8, 3, 7, and 8 participants, respectively, had mailed a low volume of hair but enough to analyze for one ARV. There were four samples that could not be analyzed due to laboratory assay failure. The distal end of the hair samples were not labeled by 9, 5, 6, and 3 participants at months 0, 2, 4, and 6, respectively.

### Acceptability

The exit survey was completed by 84 individuals, 74.2% of whom reported an excellent overall experience with the study (Table 4). The majority reported being extremely satisfied with being part of a remotely conducted research project, text messaging photographs of refill dates and pill counts to the study staff, level of support from the study staff, and the remote compensation method. Approximately 60.2% were very or extremely satisfied with participating in a hair collection study and 65.6% were very or extremely satisfied with mailing hair samples every two months. The majority reported that collecting hair samples was very or extremely easy (54.9%), using the mailed hair collection kits was very or extremely easy (72.0%), and mailing hair samples to the study was very or extremely easy (80.6%). Most participants stated extremely high likelihood of recommending a similar study to a friend (69.9%), participating in a similar study including text messaging pictures of medications or refill dates (76.3%), and participating in a similar study including mailing hair samples to study staff (59.1%).

**Table 4:** Responses to exit survey.

Assessment	Response	N = 84*
1- Rate overall experience with the study		
	Excellent	69 (74.2)
	Good	15 (16.1)
	Fair	0 (0)
	Poor	0 (0)
	Missing	9 (9.7)
2- Satisfaction with participating in remote research project		
	Extremely satisfied	69 (74.2)
	Very satisfied	15 (16.1)
	Somewhat satisfied	0 (0)
	Somewhat unsatisfied	0 (0)
	Very unsatisfied	0 (0)
	Extremely unsatisfied	0 (0)
	Missing	9 (9.7)
3- Satisfaction with participating in hair collection study		
	Extremely satisfied	36 (38.7)
	Very satisfied	20 (21.5)
	Somewhat satisfied	21 (22.6)
	Somewhat unsatisfied	7 (7.5)
	Extremely unsatisfied	0 (0)
	Missing	9 (9.7)
4- Satisfaction with mailing hair samples every 2 months		
	Extremely satisfied	40 (43.0)
	Very satisfied	21 (22.6)
	Somewhat satisfied	17 (18.3)
	Somewhat unsatisfied	6 (6.5)
	Extremely unsatisfied	0 (0)
	Missing	9 (9.7)
5- Satisfaction with taking photos of remaining medications or refill dates and text message photos to study staff		
	Extremely satisfied	60 (64.5)
	Very satisfied	18 (19.4)
	Somewhat satisfied	4 (4.3)
	Somewhat unsatisfied	2 (2.2)
	Extremely unsatisfied	0 (0)
	Missing	9 (9.7)
6- Satisfaction with security and privacy of text messages		
	Extremely satisfied	63 (67.7)
	Very satisfied	17 (18.3)
	Somewhat satisfied	2 (2.2)
	Somewhat unsatisfied	2 (2.2)
	Extremely unsatisfied	0 (0)
	Missing	9 (9.7)
7- Satisfaction with level of information and staff support		
	Extremely satisfied	64 (68.8)
	Very satisfied	15 (16.1)
	Somewhat satisfied	4 (4.3)
	Somewhat unsatisfied	1 (1.1)
	Extremely unsatisfied	0 (0)
	Missing	9 (9.7)
8- Satisfaction with use of the remote compensation method		
	Extremely satisfied	60 (64.5)
	Very satisfied	16 (17.2)
	Somewhat satisfied	5 (5.4)
	Somewhat unsatisfied	2 (2.2)
	Extremely unsatisfied	0 (0)
	No response	1 (1.1)
	Missing	9 (9.7)
9- Ease or difficulty with collecting hair		
	Extremely easy	30 (32.3)
	Very easy	21 (22.6)
	Somewhat easy	18 (19.4)
	Somewhat difficult	11 (11.8)
	Very difficult	2 (2.2)
	Extremely difficult	2 (2.2)
	Missing	9 (9.7)
10- Ease or difficulty with using mailed hair kits		
	Extremely easy	47 (50.5)
	Very easy	20 (21.5)
	Somewhat easy	13 (14.0)
	Somewhat difficult	4 (4.3)
	Very difficult	0 (0)
	Extremely difficult	0 (0)
	Missing	9 (9.7)
11- Ease or difficulty with mailing hair to study		
	Extremely easy	60 (64.5)
	Very easy	15 (16.1)
	Somewhat easy	7 (7.5)
	Somewhat difficult	2 (2.2)
	Very difficult	0 (0)
	Extremely difficult	0 (0)
	Missing	9 (9.7)
12- Ease or difficulty with taking photos of remaining medications or the refill dates		
	Extremely easy	60 (64.5)
	Very easy	14 (15.1)
	Somewhat easy	6 (6.5)
	Somewhat difficult	4 (4.3)
	Very difficult	0 (0)
	Extremely difficult	0 (0)
	Missing	9 (9.7)
13- Ease or difficulty with text messaging photos of remaining medications or refill date to study staff		
	Extremely easy	61 (65.6)
	Very easy	17 (18.3)
	Somewhat easy	4 (4.3)
	Somewhat difficult	2 (2.2)
	Very difficult	0 (0)
	Extremely difficult	0 (0)
	Missing	9 (9.7)
14- Level of helpfulness of communication with study staff		
	Extremely helpful	60 (64.5)
	Very helpful	20 (21.5)
	Moderately helpful	3 (3.2)
	A little helpful	1 (1.1)
	Not at all helpful	0 (0)
	Missing	9 (9.7)
15- Level of helpfulness of video watched on collecting hair samples before study participating		
	Extremely helpful	54 (58.1)
	Very helpful	21 (22.6)
	Moderately helpful	6 (6.5)
	A little helpful	2 (2.2)
	Not at all helpful	0 (0)
	No response	1 (1.1)
	Missing	9 (9.7)
16- Level of helpfulness of written instructions on hair collection		
	Extremely helpful	57 (61.3)
	Very helpful	20 (21.5)
	Moderately helpful	5 (5.4)
	A little helpful	2 (2.2)
	Not at all helpful	0 (0)
	Missing	9 (9.7)
17- Receive help from others in collecting hair samples		
	Yes	29 (31.2)
	No	55 (59.1)
	Missing	9 (9.7)
18- Amount of improvement in medication adherence by participating in study		
	A great deal	22 (23.7)
	A lot	14 (15.1)
	A moderate amount	13 (14.0)
	A little	10 (10.8)
	Not at all	25 (26.9)
	Missing	9 (9.7)
19- Likelihood of recommending a similar study to a friend		
	Extremely likely	65 (69.9)
	Very likely	13 (14.0)
	Somewhat likely	5 (5.4)
	Somewhat unlikely	1 (1.1)
	Very unlikely	0 (0)
	Extremely unlikely	0 (0)
	Missing	9 (9.7)
20- Likelihood of participating in a similar study including text messaging pictures of medications or refill dates		
	Extremely likely	71 (76.3)
	Very likely	10 (10.8)
	Somewhat likely	3 (3.2)
	Somewhat unlikely	0 (0)
	Very unlikely	0 (0)
	Extremely unlikely	0 (0)
	Missing	9 (9.7)
21- Likelihood of participating in a similar study including mailing hair samples to study staff		
	Extremely likely	55 (59.1)
	Very likely	13 (14.0)
	Somewhat likely	12 (12.9)
	Somewhat unlikely	2 (2.2)
	Very unlikely	1 (1.1)
	Extremely unlikely	1 (1.1)
	Missing	9 (9.7)

* Missing data on 9 participants (9.7%)

Most did not receive help from others in collecting hair samples (59.1%). There was a wide distribution of the self-perceived impact of study participation on improvement in ARV adherence (23.7% reported a great deal of improvement while 26.9% reported no improvement at all).

The qualitative exit interviews were conducted with 32 participants who met one of the pre-specified study engagement categories: (a) On Time: N = 12; (b) Early Inconsistent Hair Samples: N = 5; (c) Late Inconsistent Hair Samples: N = 5; (d) Inconsistent Texts: N = 3; and (e) Least Consistent: N = 7. We over sampled in the Least Consistent group because the Inconsistent Texts group only had three participants. Most participants stated that the study was simple, convenient, not time-consuming, and private (S1 Table). One participant noted:

“… the advantages of course being able to be at home, not having to get dressed, not having to travel… it felt more confidential and… I felt very supported and it was kinda cool using today’s technology… you guys really provided that type of support. If I had a question, if anything was up in the air, we were interacting in real time… I was happy to see you had worked out a system that really worked.” (55 year-old, African American, male)

Participants reported improvements in their awareness toward taking ARVs and a sense of pride in contributing to research. The study’s remote methodology was particularly appreciated by two participants, one with a busy schedule that would have prohibited him from participating in research and one with a disability:

“… for half of the time that I've been in the study, I haven't even been in my home city. So, it probably actually made all the difference between me being able to participate and not.” (30 year-old, White, male)

“… I'm disabled, so I'm unable to get around to other surveys like I have been… but I'm having a disability issue right now, so I'm not able to go. So I thought the telephone way was pretty convenient. And it's always on time… you guys are flexible too … It was far more easier and far more rewarding too.” (37 year-old, multiracial, transgender female)

The main disadvantages of the study were noted by participants who needed assistance with study activities (e.g., cutting hair or sending text messages) and those who missed the personal connection:

“I did not get to meet you… in person. I've been in several studies… and it's always with a person … I think that made it … more objective maybe that I didn't have any kind of emotional connection with the person… I don't know if it's positive or negative. I guess it's mixed.” (74 year-old, White, male)

Participants had differing opinions regarding the need to collect hair samples. Some found it to be easy and without hassle, while others reported challenges. One participant stated:

“I didn't give it a thought, certainly easier than giving 1,000 tubes of blood… I have a good pair of scissors and it just wasn't a difficult thing to do…. My hair is long enough so that I can do it.” (75 year-old, White, male)

While another noted:

“That was the worst part. It's very hard to do. I have very short hair and was always concerned that I didn't get enough and because we didn't do hair every month and it was very hard to know when I would be expected so that I could let it grow so that I'd have more hair to sample from. So, I think having had a schedule that… I know I'm going to get an envelope around this date to do a sample, then I could've let the hair grow a little bit longer. But there were times where I would've shaved and then the next day, the envelope would show up…” (41 year-old, White, male)

Participants discussed workarounds, such as waiting to collect their hair sample shortly before requiring a haircut or working with their barber or hairstylist to cut their hair. One participant highlighted age and cultural differences in his perceptions toward collecting hair samples:

“I was kind of hesitant at first … It’s an African American thing too about our hair… different styles of hair because African Americans, we can have so many different styles of hair and so for us I see it as art form and so I can only speak on what other people said that they felt like they couldn’t just take like a plug out of their hair. It would mess up the whole appearance of their hair. So for me, I struggled with where I could take [the hair sample] from where people wouldn’t see and then how much… The other thing because I’m at the aging point in my life… my hair was thinning on top… So for older people I think that might be a factor… ‘cause the hair they have left is so scarce…” (55 year-old, African American, male)

Similarly, another African American participant stated:

“I am an African-American woman and we do have a great sense of love for our hair. So, it was okay… because when you looked at the instructions, it's whether you have short hair or you were wearing braids. It made it easy to get the hair sample because you had different ways you could produce your hair sample as far as cutting the hair or just letting it fall out onto the… collection little foil that you had.” (50 year-old, African American, female)

Few participants had any concerns regarding privacy and security of their hair sample. One individual noted their hesitancy at the beginning of the study as such:

“[I] had some concerns. I'm like, what are you going to be using my hair for? I had a whole bunch of questions. But once you explained it to me, what you guys were doing, I was all right with it. Because at first, I was like, I've got to send my hair in? Where is it going? Who's going to be looking at it? Why is it being used? But you answered all the questions, which is why I went ahead and participated in the study.” (48 year-old, White, male)

The remote compensation method was appreciated by all participants. Fourteen individuals reported lost or stolen compensation debit cards resulting in a need to wait for replacements cards. However, overall, participants stated that they liked this method better than gift cards for specific vendors and viewed it as a preferred method for research compensation, as one reported:

“I think that’s the way any kind of compensation in regards to any kind of research should be done… at the end of the month after each, after each submission, I said ‘oh yea I have a little extra cash, to do something with it’… especially for someone who found themselves kind of strapped financially that was really helpful.” (56 year-old, Asian, male)

The main issues around text messaging photographs of medications or refill dates that were brought up by participants were around the inconvenience of having to take their medications out of the vial, disconnected mobile telephone services, or needing to learn how to use their telephone, as stated by one participant:

“It was pretty much easy… I'm old-fashioned… so… I got multiple learning out of the whole thing how to use my phone… (57 year-old, African American, male)

When asked about the frequency of text messages, most participants reported that the communications were not bothersome and, in fact, could have increased in frequency. This was captured by two participants as such:

“… as I think about it, it was pretty perfect. It wasn't too much and it wasn't too [little]. But again what I think would have helped me is like a reminder like: ‘This week… we'll do the survey'… I would probably have liked a text reminder… And I actually appreciated the text, it's just easier than email… Or even giving people the option. Some people prefer email some people prefer text.” (55 year-old, African American, male)

“… another text, or a phone call… we sent out the kit and it should be there Wednesday or Thursday or something. And then, I would have specifically gone to look for it ‘cause if I wasn't expecting something… then nothing's coming. I'm not getting any mail. So either a phone call or a text or something that says… either it should be there already or you might want to check… tomorrow and the next day.” (47 year-old, White, male)

Some participants did not notice any change in their ARV adherence due to already high adherence; however, some noted an improvement in taking medications or being more aware of their medications. This was captured by one participant:

“Well, it just adds an extra incentive for me to stay on top of it… I’m already being rewarded with good health, but to be able to be paid for staying on top of my adherence, yes, that speaks volumes. And it's actually… habitual, it's a way to develop a habit of taking better care of yourself. Because even if you're not getting compensation… just having had gone through this process, it just gives you a better awareness of your pills you take, and how much you have, and where to store them, and how to store them.” (37 year-old, multiracial, transgender female)

## Discussion

We examined the feasibility and acceptability of conducting an entirely remote study using three novel measures of ARV adherence among a diverse sample of participants nationwide. We noted high levels of feasibility and acceptability with the overall study methodology, remote data collection, collection of adherence data using photographed and text messaged pharmacy refill dates and pill counts, and home-collected hair samples. Despite some individuals requiring additional assistance with study activities and reporting that they missed the personal connection, the majority of participants noted that the study was simple, convenient, not time-consuming, and private. While many reported an improvement in their ARV adherence, several individuals noted no change due to an already high level of adherence. To our knowledge, this is the first study among PLWH that has been conducted in a completely remote manner and examined remote collection of refill dates, pill counts, and hair samples.

The home-collection of hair samples to estimate ARV medication adherence is a novel concept and holds much promise for future HIV treatment and prevention research[17]. Hair is easy to collect, is non-invasive, can be stored at room temperature, and does not require biohazardous shipping precautions. These features all render hair an ideal biomatrix for home-collection. Medication concentrations in hair reflect drug uptake from the systemic circulation over weeks to months and provide an average measure of ARV medication exposure[18, 19]. Disadvantages to measuring adherence based on home-collected hair samples include the time required between mailing out hair kits to participants and receipt of hair samples as well as the time required to measure ARV concentrations using LC-MS/MS. Feedback from the laboratory conducting the assays (i.e., the UCSF Hair Analytical Laboratory) regarding low sample volume was helpful for us to notify each person individually about improving hair collection, even though this only affected the hair concentrations for one sample. This feedback may be particularly helpful in other studies if more than on ARV concentration is being analyzed per hair sample and therefore a larger sample volume is required. Additionally, we have developed a hair collection video and step-by-step instructions for research participants to minimize challenges and concerns related to hair collection (rxpix.ucsf.edu).

In a preliminary formative study, our team reported a high degree of correlation and agreement between hair samples collected from the side of the head (a region easier to reach for home-collection) and hair concentrations collected from the back of the head (an area from which research samples are typically collected)[17]. Additionally, we showed a high degree of correlation and agreement between ARV levels in hair collected by trained staff and at home by participants without any evidence of measurement bias[17]. We have further expanded this research on home-collection of hair in the current pilot study by using only remotely-collected hair samples from a larger group of PLWH nationwide.

Photographed and text messaged pharmacy refill dates from the medication vial to estimate refill-based adherence and photographed medication quantity to calculate pill-count-based adherence are novel approaches to estimate ARV adherence. When using pharmacy-collected refill records and in-person or clinic-based pill counts, these methods have offered advantages and disadvantages. Both are reliable, valid, and objective measure of adherence [7, 20–22]. When collected via photographed text messages, there is a potential for time-saving due to not having to contact the participant’s pharmacy (if conducting research outside of a closed healthcare systems, linking a patient’s electronic medical record to pharmacy data) or conducting phone-based unannounced pill counts. Additionally, both can be collected with little financial resources in a wide range of clinic and research settings. However, we encountered some difficulties with participants who had a stock supply of medications, used medication vials with out-of-sequence dates, had higher pill counts than the last remotely-conducted study visit without having received a new refill, received a 60- or 90-day ARV refill, or discarded the medication box containing the pharmacy label prior to the study visit. These scenarios posed problems due to our inability to accurately capture adherence. We believe that some of these problems can be resolved with real-time adherence calculations that can alert study staff of illogical data.

The only feasibility metric that we did not meet was the mean number of reminder text messages prior to response. We had pre-specified a total of two reminder text messages per enrolled participant, but in reality, we needed a mean of 2.6 reminders per participant. This information along with the responses from the qualitative interviews noting that the frequency of communications was not bothersome indicates the need for more frequent reminders and communication. Prior research has also indicated that weekly or less frequent text messaging is associated with greater intervention efficacy[23]. Other lessons learned included the need for text messages to participants upon mailing out of the hair kits and overall study schedule, clearer guidance regarding how to replace lost or stolen compensation debit cards, and more technical assistance for those with disconnected mobile telephone services or those unfamiliar with their phone technology. As with any research, there are limitations to our pilot study. Even though we used a multi-pronged approach to recruit participants, social media was the prominent method used. Likewise, the methods required using text messaging to send messages and pictures and to complete surveys online; thus, the sample had at least a minimal comfort with technology. Additionally, many participants had a high level of ARV adherence and reported having an undetectable HIV viral load. Therefore, these data may not be generalizable to individuals who are not social media users, are less technologically savvy, or are more engaged in their HIV care. Finally, we took a practical approach to the qualitative component of the acceptability evaluation, in which we used the interview guide as the structure for analysis and presentation of results. A more formal analytic approach may have provided greater rigor to the methods; however, our targeted approach sampled participants across a range of experiences with the study and promoted specific inquiry about the full scope of study methods whose acceptability were of interest.

Using a remote methodology, we were able to recruit participants from Western, Midwestern, Southern, and Northeastern regions of the US. Participants had high levels of satisfaction with the remote manner of the study and found it be convenient, not time-consuming, and private. Two participants specifically commented on their inability to participate in non-remote research due to disability and a busy schedule. Based on one participant, the remote nature of this study may have created a more “objective” environment which may have enhanced the accuracy of collected data. Therefore, our research expands the reach of research participation to individuals whose perspectives may otherwise have been missed due to reasons such as stigma, remote location, transportation barriers, disability, and busy schedules[12]. Future research is needed to determine how well these remote adherence assessment approaches compare to other methods in capturing adherence over time, measuring the impact of adherence-enhancing interventions, and predicting virologic suppression over time.

In summary, this is the first study to examine an entirely remote methodology of three novel measures to assess ARV adherence among PLWH. Participants reported high satisfaction with the various research components and the three adherence measures were feasible and acceptable to collect. All measures were objective, easy to collect, relatively cost-effective, and able to be collected remotely. These adherence measures represent innovative approaches to expand monitoring tools for HIV treatment and prevention adherence in future research.

## Supporting information

S1 Table (DOCX)
