# Supplementary material for: Feasibility and acceptability of novel methods to estimate antiretroviral adherence: A longitudinal study
Source: PLoS One. 2019 Jan 15;14(1):e0210791. doi: 10.1371/journal.pone.0210791 (PMC6333375; doi:10.1371/journal.pone.0210791)
Supplement: S1 Table — (DOCX) [file pone.0210791.s001.docx]

Supporting Information

**S1 Table: RxPix Qualitative Analysis Themes and Exemplary Quotes**

| **Question** | **Theme** | **Quotes** |
| --- | --- | --- |
| How did you feel about participating in a research project that was conducted without you having to come into a clinic or research site (i.e., remotely)? What were some advantages and disadvantages of this approach? | Overall Impression of Remote Study | - “… the advantages of course being able to be at home, not having to get dressed, not having to travel… it felt more confidential and… I felt very supported and it was kinda cool using today’s technology… you guys really provided that type of support. If I had a question, if anything was up in the air, we were interacting in real time… I was happy to see you had worked out a system that really worked.”  - “… for half of the time that I've been in the study, I haven't even been in my home city. So, it probably actually made all the difference between me being able to participate and not.”  - “… I'm disabled, so I'm unable to get around to other surveys like I have been… but I'm having a disability issue right now, so I'm not able to go. So I thought the telephone way was pretty convenient. And it's always on time… you guys are flexible too... It was far more easier and far more rewarding too.”  - “I did not get to meet you… in person. I've been in several studies… and it's always with a person... I think that made it... more objective maybe that I didn't have any kind of emotional connection with the person… I don't know if it's positive or negative. I guess it's mixed.” |
| What did you think about receiving compensation for participating in the study with the ClinCard? | Receiving Remote Compensation Using Clincard | - “I think that’s the way any kind of compensation in regards to any kind of research should be done… at the end of the month after each, after each submission, I said ‘oh yea I have a little extra cash, to do something with it’… especially for someone who found themselves kind of strapped financially that was really helpful.” |
| What did you think about collecting your hair sample for the study?  a. What problems did you face? How did you overcome them?  b. How was it collecting the hair yourself / with help from someone? | Thoughts about Collecting Hair Samples | - “I didn't give it a thought, certainly easier than giving 1,000 tubes of blood… I have a good pair of scissors and it just wasn't a difficult thing to do…. My hair is long enough so that I can do it.”  - “That was the worst part. It's very hard to do. I have very short hair and was always concerned that I didn't get enough and because we didn't do hair every month and it was very hard to know when I would be expected so that I could let it grow so that I'd have more hair to sample from. So, I think having had a schedule that… I know I'm going to get an envelope around this date to do a sample, then I could've let the hair grow a little bit longer. But there were times where I would've shaved and then the next day, the envelope would show up…”  - “I was kind of hesitant at first... It’s an African American thing too about our hair… different styles of hair because African Americans, we can have so many different styles of hair and so for us I see it as art form and so I can only speak on what other people said that they felt like they couldn’t just take like a plug out of their hair. It would mess up the whole appearance of their hair. So for me, I struggled with where I could take [the hair sample] from where people wouldn’t see and then how much… The other thing because I’m at the aging point in my life… my hair was thinning on top… So for older people I think that might be a factor… ‘cause the hair they have left is so scarce…” |
| Were there times that you did not mail your hair to the study? If so, why? | Late/No Hair Samples | - “… another text, or a phone call… we sent out the kit and it should be there Wednesday or Thursday or something. And then, I would have specifically gone to look for it ‘cause if I wasn't expecting something… then nothing's coming. I'm not getting any mail. So either a phone call or a text or something that says… either it should be there already or you might want to check… tomorrow and the next day.” |
| Did you ever collect hair from a place other than the side of your head? If so was why? | Non Head Hair | Not reported by participants |
| How easy or difficult was it to take photos of your medication refill dates and pill counts and text them to the study phone number? | Texting Photos of Meds and Refill Dates | - “It was pretty much easy… I'm old-fashioned… so… I got multiple learning out of the whole thing how to use my phone… |
| What are your thoughts around the cultural appropriateness of the study procedures? | Cultural Appropriateness | - “[I] had some concerns. I'm like, what are you going to be using my hair for? I had a whole bunch of questions. But once you explained it to me, what you guys were doing, I was all right with it. Because at first, I was like, I've got to send my hair in? Where is it going? Who's going to be looking at it? Why is it being used? But you answered all the questions, which is why I went ahead and participated in the study.”  - “I am an African-American woman and we do have a great sense of love for our hair. So, it was okay… because when you looked at the instructions, it's whether you have short hair or you were wearing braids. It made it easy to get the hair sample because you had different ways you could produce your hair sample as far as cutting the hair or just letting it fall out onto the… collection little foil that you had.” |
| Did you experience any technical issues that made it difficult to respond to the monthly text surveys? | Technical Issues | No major technical issues reported. |
| How did you feel about the frequency of texts from the study team? | Text Frequency | - “… as I think about it, it was pretty perfect. It wasn't too much and it wasn't too [little]. But again what I think would have helped me is like a reminder like: ‘This week… we'll do the survey'… I would probably have liked a text reminder… And I actually appreciated the text, it's just easier than email… Or even giving people the option. Some people prefer email some people prefer text.” |
| How do you think participating in this study has changed the way you take your medications? What differences have you noticed before and after this study? | Change in Adherence | - “Well, it just adds an extra incentive for me to stay on top of it… I’m already being rewarded with good health, but to be able to be paid for staying on top of my adherence, yes, that speaks volumes. And it's actually… habitual, it's a way to develop a habit of taking better care of yourself. Because even if you're not getting compensation… just having had gone through this process, it just gives you a better awareness of your pills you take, and how much you have, and where to store them, and how to store them.” |
